# Primary intradural extramedullary extraosseous Ewing’s sarcoma/peripheral primitive neuroectodermal tumor (PIEES/PNET) of the thoracolumbar spine: A case report and literature review

**DOI:** 10.1515/med-2021-0377

**Published:** 2021-10-21

**Authors:** Feifei Pu, Jianxiang Liu, Zhicai Zhang, Tao Guo, Zengwu Shao

**Affiliations:** Department of Orthopedics, Union Hospital, Tongji Medical College, Huazhong University of Science and Technology, Wuhan 430022, China; Department of Pathology, Union Hospital, Tongji Medical College, Huazhong University of Science and Technology, Wuhan 430022, China; Department of Orthopedics, Union Hospital, Tongji Medical College, Huazhong University of Science and Technology, No. 1277, Jiefang Avenue, Jianghan Distinct, Wuhan 430022, China

**Keywords:** chemotherapy, PIEES/PNET, radiotherapy, surgical resection, thoracolumbar

## Abstract

We present a rare case of a primary intradural extramedullary Ewing’s sarcoma/peripheral primitive neuroectodermal tumor (PIEES/PNET) in the thoracolumbar spine and review the current literature. We describe the imaging manifestations, pathological features, surgical methods, and patient survival to shed light on the clinical management of this rare tumor. A 32-year-old man experienced progressive low back pain for more than 1 month. An intradural extramedullary tumor from T12 to L2 was detected on magnetic resonance imaging. He underwent a thoracolumbar laminotomy for decompression, complete excision of the intradural extramedullary tumor, and internal fixation with pedicle screws. A histopathological examination confirmed that the tumor was a PIEES/PNET via an immunohistochemical study of the surgically resected tissues. Postoperatively, the patient received chemotherapy and radiotherapy. No recurrence, metastasis, or failure of internal fixation were noted at a 17-month post-surgery radiographic examination. PIEES/PNET of the thoracolumbar spine is extremely rare. Treatment is difficult because the current literature is sparse and cases are rare. Complete resection combined with chemotherapy and radiotherapy effectively reduces recurrence and metastasis.

## 1 Introduction

Ewing’s sarcoma/peripheral primitive neuroectodermal tumor (ES/PNET) is a mesenchymal tumor characterized by small round blue cells, poor differentiation, and high malignancy [1]. ES/PNET is among the most common primary malignant bone tumors in children. ES/PNET generally affects the long bones and, more rarely, the spine (3.5% of cases) [2]. Specifically, the incidence of primary intradural extramedullary ES/PNET (PIEES/PNET) of the spine is extremely rare [3]. To date, only a few cases in the thoracolumbar spine have been reported in the literature (Table 1) [4,5,6,7,8,9,10,11,12,13]. Here we describe a rare case of PIEES/PNET in the thoracolumbar spine and review the literature.

**Table 1:** Literature review of cases of PIEEES/PNET of thoracolumbar

Author/year	Age/gender	Signs/symptoms	Duration of symptoms	Levels	Resection	Adjuvant therapy	Long-term outcomes
Hisaoka et al. 1997 [4]	14/male	Low back pain	3 months	T12–L1	Gross total resection	N/A	Well without evidence of disease at 3 months after the surgery
Haresh et al. 2008 [5]	26/male	Lower limb weakness	2 months	T11–S2	Gross total resection	Chemotherapy (VCR, ADM, CTX) and radiation (5,000 cGy)	Clinically stable at 6 months after treatment
Jia et al. 2009 [6]	28/male	Lower limb weakness	15 days	T12–L3	Gross total resection		Sarcoma recurred and metastasis at 9 months after the operation
Vincentelli et al. 2010 [7]	40/female	Lower limb weakness	1 week	T11–L4	Gross total resection	Chemotherapy (ADM, IFO) and radiation (4,000 cGy)	The conditions were good at 6 months after treatment
Ellis et al. 2011 [8]	35/male	Low back pain	6 months	T12–L2	Subtotal resection	N/A	No revealed metastases or possible primary lesions at 2 months following surgery
Mardekian et al. 2014 [9]	26/male	Low back pain	N/A	T12–L1	Gross total resection	N/A	N/A
	70/male	Low back pain	N/A	T12–L1	Subtotal resection	N/A	N/A
Mateen et al. 2011 [10]	50/male	Lower limb weakness	3 months	T11–L1	Subtotal resection	Chemotherapy (VCR, CTX, ADR, IFO) and radiation (5,040 cGy)	Died with diffuse disease limited to the nervous system at 48 months after initial diagnosis
Chihak et al. 2016 [11]							
Yan et al. 2019 [12]	60/male	Low back pain and incontinence	1 month	T12–L3	Subtotal resection	Chemotherapy	N/A
Izubuchi et al. 2020 [13]	35/female	Low back pain and lower limb weakness	2 months	T12–L1, L4–5	Subtotal resection	Chemotherapy (VDC, IE) and radiotherapy (total dose of 45 Gy)	Died of diffusely disseminated disease limited to the central nervous system at 16 months after the initial diagnosis
Current study	32/male	Low back pain and lower limb weakness	1 month	T12–L2	Gross total resection	Chemotherapy (CTX, THP, VCR, IFO, VP-16) and radiation (5,000 cGy)	Disease free at 88 months

N/A: no information available; VCR: vincristine; ADM: doxorubicin; CTX: cyclophosphamide; IFO: ifosfamide; ADR, adriamycin; VDC: vincristine, doxorubicin, and cyclophosphamide; IE: ifosfamide and etoposide; THP: pirarubicin; VP-16: etoposide.

## 2 Case presentation

A previously healthy 32-year-old man experienced progressive low back pain for more than 1 month. Physical examination showed percussive pain in the thoracolumbar spinous process, but no significant mass was detected. The muscle strength of the right lower limb was grade 4, while that of the left lower limb was grade 5. The right anterolateral thigh felt numb, while the left side was normal. The Lasegue test was positive on the right side and negative on the left. Bilateral Achilles tendon, knee reflexes, and Babinski sign were negative.

**Figure 1:**
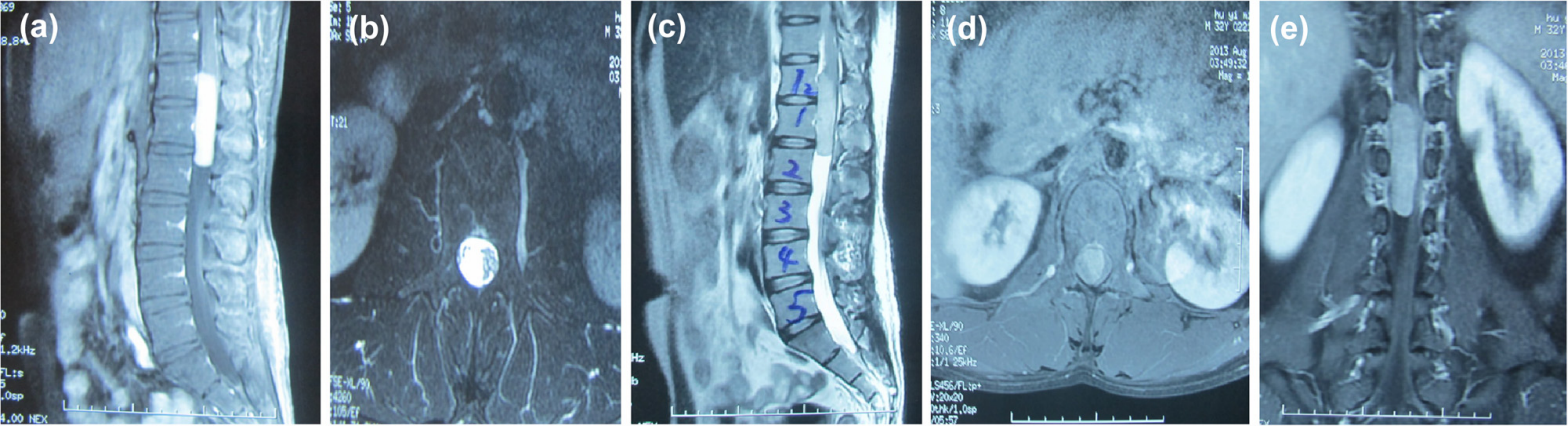
Spinal MRI examination revealed a circular mass in the spinal canal of T12–L2 with unclear boundaries. (a and b) Signaling within the tumor was not uniform, with hypointensity on T1-weighted images (T1WI) and (c–e) speckled hyperintensity on T2-weighted images (T2WI).

Spinal magnetic resonance imaging (MRI) revealed a circular mass in the spinal canal of T12–L2 with unclear boundaries. Signaling within the tumor was not uniform, with hypointensity on T1-weighted imaging (T1WI) (Figure 1a and b) and speckled hyperintensity on T2-weighted imaging (T2WI) (Figure 1c–e).

**Figure 2:**
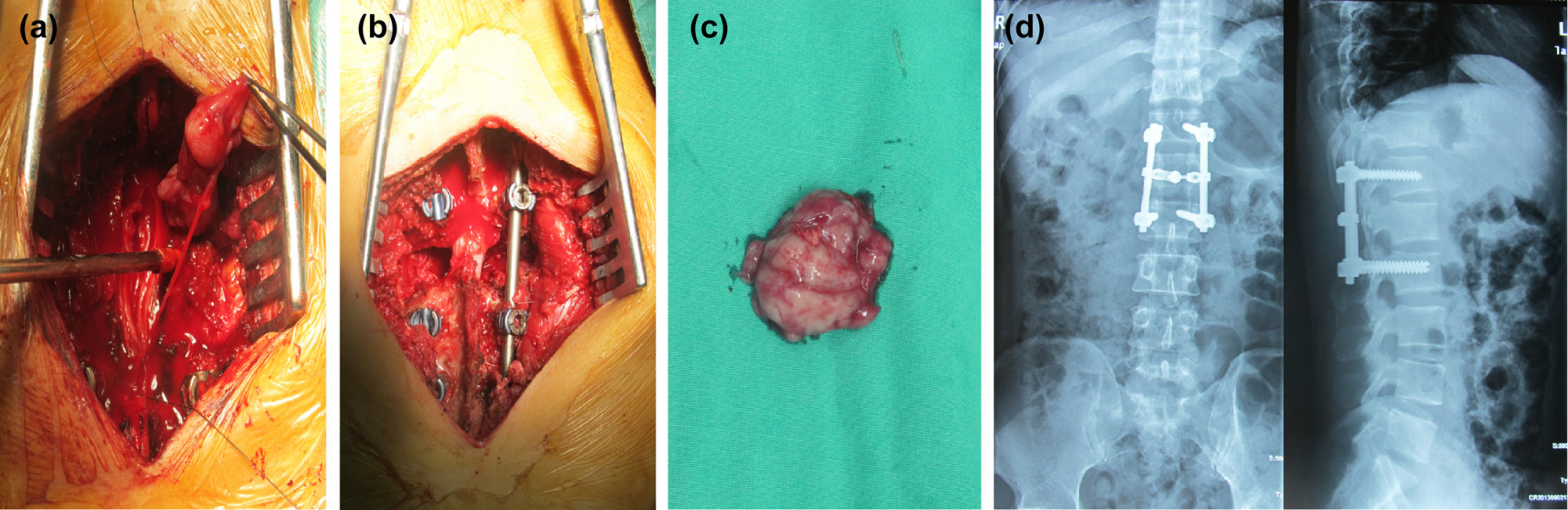
(a) The nerve roots were carefully separated from the tumor under a microscope and the tumor was completely excised. (b) There was no residual tumor in the spinal canal. (c) The fish-shaped round mass obtained by gross total resection. (d) Pedicle screws and connecting rods were used to reconstruct the area.

The patient underwent thoracolumbar spinal canal tumor resection under general anesthesia. The lamina and spinous process were excised with an ultrasonic bone knife and the dural sac was gently incised with a sharp knife to expose the spinal canal. Intraoperatively, a quasi-round tumor was observed in the spinal canal of T12–L2. The tumor was red and fish-like with a soft texture. The tumor’s capsule was incomplete, its boundary was unclear, and it was adherent to the peripheral nerve roots. The nerve roots were carefully separated from the tumor under a microscope, and the tumor was completely excised (Figure 2a). We confirmed that there was no residual tumor in the spinal canal (Figure 2b and c). Four pedicle screws were placed on the T12 and L1 pedicles, and two connecting rods were used to reconstruct the vertebral body (Figure 2d).

**Figure 3:**
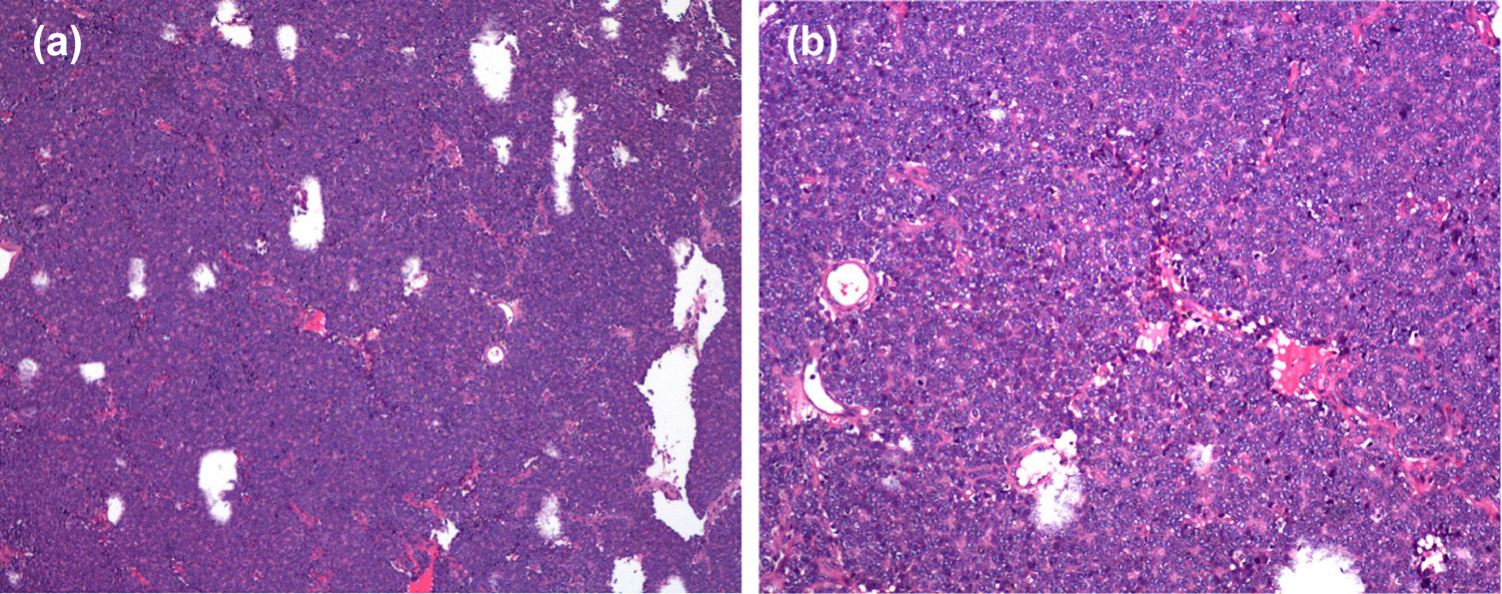
Postoperative pathology of the tumor tissue revealed undifferentiated small round blue cells with hematoxylin and eosin staining: ×40 (a) and ×100 (b).

Immunohistochemistry revealed: Fli-1 (+), Vim (+), CD56 (+), CD99 (+), Syn (+), weak focal S-100, weak focal CD117, PCK (−), TdT (−), LCA (−), EMA (−), CgA (−), CD34 (−), MyoD1 (−), desmin (−), and HMB45 (−). Postoperative pathology of the tumor tissue revealed undifferentiated small round blue cells, and the pathological diagnosis was PIEES/PNET (Figure 3a and b). On postoperative day 3, the patient’s pain and numbness in the waist and lower extremities were relieved. Postoperatively, the patient received 4 cycles of chemotherapy and 12 rounds of radiotherapy. The chemotherapy regimen was CAV(CTX + THP + VCR)/IE(IFO + VP-16), while the radiotherapy dose was 5000 cGy. No recurrence, metastasis, or failure of internal fixation was noted as of a 17-month postoperative radiographic examination. However, the patient was lost to follow-up, and we are unaware of the subsequent outcome.

The study was approved by the Ethics Committee of Union Hospital, Tongji Medical College, Huazhong University of Science and Technology. The patient provided informed consent for the publication of this case report.

## 3 Discussion

PNET is a rare and highly malignant tumor of the nervous system and a member of the Ewing’s sarcoma family of tumors. PNET, more common in children and adolescents, has differentiation potential and a short disease course, and mainly occurs in the paraspinal and retroperitoneal areas and the extremities, whereas intraspinal PNET is rare [14]. The main symptoms of intraspinal PNET include sensory and movement disturbances, which lead to decreased muscle strength, decreased tendon reflexes, hypoesthesia, and positive pathological signs [15]. However, these clinical manifestations are difficult to distinguish from other intraspinal tumors and are easily ignored or misdiagnosed as other diseases in the early stages of intraspinal PNET. In this case, the patient experienced progressive low back pain, and the physical examination showed percussive pain in the thoracolumbar spinous process. The patient reported decreased sensation and muscle strength in the right lower limb, and the Lasegue test result was positive. PIEES/PNET is highly malignant with a rapidly progressing course [16]. Diagnosing the patient in this case took only 1 month from the onset of the disease. Therefore, when children or adolescents are mainly characterized by a progressive decline in muscle strength, imaging suggests space-occupying lesions in the spinal canal, and the disease progresses rapidly, the possibility of PIEES/PNET should be considered.

At present, imaging of PIEES/PNET mainly involves MRI, and it often presents as a single mass in the spinal canal and extramedullary space [12,13]. MRI shows isosignal intensity on T1WI, isosignal or hypersignal intensity on T2WI, and enhanced signaling after an enhanced scan [17]. Cystic changes and surrounding bone destruction are seen in some cases [18]. PIEES/PNET is difficult to differentiate from schwannoma or neurofibroma on imaging alone, and the diagnosis is usually determined by postoperative histopathology. However, some patients with schwannomas or neurofibromas have dumbbell-like tumors that cross the foramen and progress slowly. In this case, the symptoms were significantly aggravated within 1 month, indicating the possibility of malignancy.

The gold standard for the diagnosis of PIEES/PNET depends on pathological examination. On gross examination, PIEES/PNET has usually a fish-like gray-red mass appearance. Microscopically the tumor is composed of large irregular sheets of small round cells divided by strands of fibrous tissue. Homer Wright pseudorosettes are a frequent finding [19]. In this case, the fish-shaped round mass was adherent to the peripheral nerve roots. Microscopically, small round blue cells were observed, consistent with literature reports. However, small round blue cells are not specific for the diagnosis of PIEES/PNET; thus, it should be distinguished from neuroblastoma, lymphoma, and rhabdomyosarcoma. The diagnosis of PIEES/PNET is further supported by molecular studies. For this reason, the diagnosis of PIEES/PNET should be confirmed by cytogenetic or molecular studies. The most common mutation occurring in 80–90% of ES/PNET is the reciprocal translocation t(11;22)(q24;q12) of the EWSR1 gene on chromosome 22 with the FLI1 gene on chromosome 11 creating the EWS/Fli-1 fusion gene [20]. The most commonly used clinical diagnostic criteria are positive expression of CD99 and positive expression of two or more different neural markers (such as NSE, Syn, S-100, Vim, and NF) [21]. In addition, negative expression of LCA and labeled myogenic tumors (Myosin) may rule out lymphoma and small round cell myogenic tumors [22]. The tumor immunohistochemical markers of this patient were as follows: Fli-1 (+), Vim (+), CD99 (+), CD56 (+), Syn (+), weak focal S-100, weak focal CD117, PCK (−), TdT (−), LCA (−), EMA (−), CgA (−), CD34 (−), MyoD1 (−), desmin (−), and HMB45 (−). The above immunohistochemical results were consistent with the diagnosis of PIEES/PNET.

PIEES/PNET is a systemic disease. It currently has no unified treatment, and treatment consisting of surgery, local high-dose radiotherapy, and chemotherapy is generally advocated [11]. Surgical removal of the tumor can effectively relieve the symptoms of spinal cord compression, but complete removal is difficult, so postoperative tumor recurrence and distant metastasis are likely [23]. Large-dose local radiation therapy is highly effective for non-metastatic PIEES/PNET [11,23]. However, radiotherapy has serious adverse effects on children’s growth and development and may even further damage the spinal cord; therefore, it should be used with caution [11]. Central PNET can be disseminated in the nervous system through the cerebrospinal fluid, and postoperative whole-axis chemotherapy should be administered. The chemotherapy regimen for peripheral PNET is mostly the same as that for ES, all data of which have been reported in individual cases, and most commonly using CAV/IE [23]. Although the incidence of PIEES/PNET is low, it is highly malignant and aggressive, with a poor prognosis and high mortality [2,3]. In this case, the patient received 4 cycles of postoperative chemotherapy and 12 cycles of postoperative radiotherapy. The chemotherapy regimen was CAV/IE, and the radiotherapy dose was 5,000 cGy. Because of surgery and adjuvant chemoradiotherapy, the patient not only had a significant reduction in spinal cord compression symptoms but also achieved satisfactory limb function and survival duration was increased. At the last follow-up at postoperative 17 months, there was no recurrence, metastasis, or failure of the internal fixation. However, the patient was then lost to follow-up; thus, we have no information about his subsequent outcome.

In summary, PIEES/PNET is a malignant tumor originating from the neuroectoderm. It grows rapidly and is difficult to diagnose early, which depends on histological and immunohistochemical examination. PIEES/PNET progresses rapidly and has a poor prognosis; thus, its treatment regimens require improvement. We reported this case to improve clinicians’ understanding of PIEES/PNET, improve its preoperative diagnosis, reduce misdiagnosis, strive for early diagnosis and treatment, and improve patient prognosis.

## Abbreviations

PIEES/PNET: primary intradural extramedullary Ewing’s sarcoma/peripheral primitive neuroectodermal tumor
ES/PNET: Ewing’s sarcoma/peripheral primitive neuroectodermal tumor
MRI: magnetic resonance imaging
T1WI: T1-weighted imaging
T2WI: T2-weighted imaging
